# The role of circulating cytokines in heart failure: a bidirectional, two-sample Mendelian randomization study

**DOI:** 10.3389/fcvm.2024.1332015

**Published:** 2024-10-22

**Authors:** Haoran Zheng, Xinxin Mao, Zhenyue Fu, Chunmei Chen, Jiayu Lv, Yajiao Wang, Yuxin Wang, Huaqin Wu, Yvmeng Li, Yong Tan, Xiya Gao, Lu Zhao, Xia Xu, Bingxuan Zhang, Qingqiao Song

**Affiliations:** ^1^ General Internal Medicine Department, Guang'anmen Hospital, China Academy of Chinese Medical Sciences, Beijing, China; ^2^ Institute of Basic Research in Clinical Medicine, China Academy of Chinese Medical Sciences, Beijing, China

**Keywords:** cytokines, heart failure, Mendelian randomization, genetics, bidirectional, two-sample

## Abstract

**Background:**

Cytokines play a pivotal role in the progression of heart failure (HF) by modulating inflammatory responses, promoting vasoconstriction, and facilitating endothelial injury. However, it is now difficult to distinguish the causal relationship between HF and cytokines in observational studies. Mendelian randomization (MR) analyses of cytokines probably could enhance our comprehension to the underlying biological processes of HF.

**Methods:**

This study was to explore the correlation between 41 cytokines with HF at the genetic level by MR analysis. We selected a HF dataset from the Heart Failure Molecular Epidemiology for Therapeutic Targets (HERMES) 2018 and a cytokine dataset from a meta-analysis of cytokine levels in Finns. Two-sample, bidirectional MR analyses were performed using Inverse Variance Weighted (IVW), Weighted Median and MR- egger, and the results were tested for heterogeneity and pleiotropy, followed by sensitivity analysis.

**Results:**

Genetic prediction of high levels of circulating Macrophage inflammatory pro-tein-1β(MIP-1β) (*P* = 0.0389), Interferon gamma induced protein 10(IP-10) (*P* = 0.0029), and Regu-lated on activation, normal T cell expressed and secreted(RANTES) (*P* = 0.0120) expression was associated with an elevated risk of HF. HF was associated with the increased levels of circulating Interleukin-2 receptor, alpha subunit(IL-2ra) (*P* = 0.0296), Beta nerve growth fac-tor(β-NGF) (*P* = 0.0446), Interleukin-17(IL-17) (*P* = 0.0360), Basic fibroblast growth factor(FGF-basic) (*P* = 0.0220), Platelet derived growth factor BB(PDGF-BB) (*P* = 0.0466), and Interferon-gamma(IFN-*γ*) (*P* = 0.0222); and with decreased levels of Eotaxin (*P* = 0.0133). The heterogeneity and pleiotropy of the cytokines were acceptable, except for minor heterogeneity of FGF-basic and IL-17.

**Conclusion:**

These findings provide compelling evidence for a genetically predictive relationship between cytokines and HF, emphasizing a great potential of targeted modulation of cytokines in slowing the progression of HF. This study draws further conclusions at the genetic level, providing a basis for future large-scale clinical trials.

## 1 Introduction

Heart failure (HF) is a group of clinical syndromes characterized by the reduction of cardiac pumping and/or congestion function (1). On a global scale, approximately 64.3 million individuals grappled with HF in 2017 (2). Those afflicted with HF face a mortality rate exceeding 30% (3) and a re-hospitalization rate surpassing 50% (4) within the initial year of diagnosis. With the aging of the population, it is estimated that the prevalence and treatment expenses of HF are anticipated to surge, presenting both medical and economic challenges worldwide. Predictions indicate that by 2030, over 80,000 individuals in the United States will contend with HF, and the direct healthcare costs for HF treatment will escalate to $5.3 billion (5). So far, HF has turned out to be a systemic syndrome that afflicts all mankind, which means that its pathophysiology needs further exploration and must be accelerated.

Ever since Levine and his team reported the elevated levels of tumor necrosis factor (TNF) in the plasma of HF patients in 1990 (6), cytokines have played a crucial role in the pathophysiological research of HF. According to the cytokine hypothesis, cytokines are responsible for the progression of HF, and the cascade of cytokine activation following myocardial injury has a detrimental impact on the circulation (7). There are 2 classes of cytokines that have been identified to play a role in HF: vasoconstrictor cytokines and vasodepressor proinflammatory cytokines (7). Cytokines exert their effects through direct membrane action or by binding to specific receptors on the cell surface (8), and then modulating inflammatory responses, promoting vasoconstriction, and inducing endothelial damage to influence the progression of HF (9). In HF patients, inadequate tissue perfusion and hypoxia lead to excessive peripheral cytokine production, activating the inflammatory and immune systems (7, 10); elevated end-diastolic wall stress in the left ventricle results in excessive expression of myocardial cytokines, directly or indirectly affecting ventricular contractile function and remodeling, thereby exacerbating HF (11). A comprehensive study involving 29 cohorts revealed a close correlation between cytokines such as interleukin-6 (IL-6), IL-18, tumor necrosis factor-alpha (TNF-α) with the onset of HF, exhibiting an approximately logarithmic-linear relationship (12). Research suggests (13) that intravenous administration of TNF*α* can induce progressive left ventricular dilation in rats. P. Aukrust and colleagues (14) identified elevated levels of monocyte chemoattractant protein-1 (MCP-1), macrophage inflammatory protein-1 alpha (MIP-1α), and RANTES in patients with chronic HF, with MCP-1 and MIP-1*α* levels significantly negatively correlated with left ventricular ejection fraction (LVEF). However, the majority of studies investigating HF and cytokines have relied on observational research designs. Yet, observational study designs face challenges in deducing the directional flow of causality and distinguishing confounding factors. Additionally, clinical trials that intervene in cytokines are also challenging. Therefore, the causal sequence between cytokines and HF progression remains a topic for further consideration. Existing unidirectional MR studies on cytokines and HF (15, 16) have primarily focused on the upstream effects of cytokines, showing different directions from clinical practice and experimental research. Therefore, the hypothesis of a relationship between HF and cytokines urgently requires evidence at the genetic level, which means incorporating more types of cytokines and assessing their impact on the disease. Studies of the genetic prediction of HF and cytokines can help to shift clinical practice from treatment to prevention for at-risk populations primarily.

Compared to observational studies, Mendelian randomization (MR) is a natural randomized statistical method using genetic variations (single nucleotide polymorphisms, SNPs) as instrumental variables (IVs) (17). Since genetic variation occurs randomly, the random assignment of alleles from parent to offspring and the unidirectional flow from genotypes to phenotypes determine the randomness of genetic variation. This process is similar to the random assignment of control and treatment groups in a population (18). Under these random conditions, MR methods can be employed to estimate the directed effects of exposures on outcomes. MR has achieved significant success in exploring disease mechanisms, identifying potential biomarkers, and targeting treatments. Numerous studies have confirmed the reliability of MR in identifying genetic relationships between exposure factors and outcomes (19–21). In this study, we used a two-sample, bidirectional MR approach to investigate the relationship between inflammatory cytokines and HF, aiming to gain insight into the role of cytokines in the onset and progression of HF.

## 2 Methods

### 2.1 Study design

**Figure 1:**
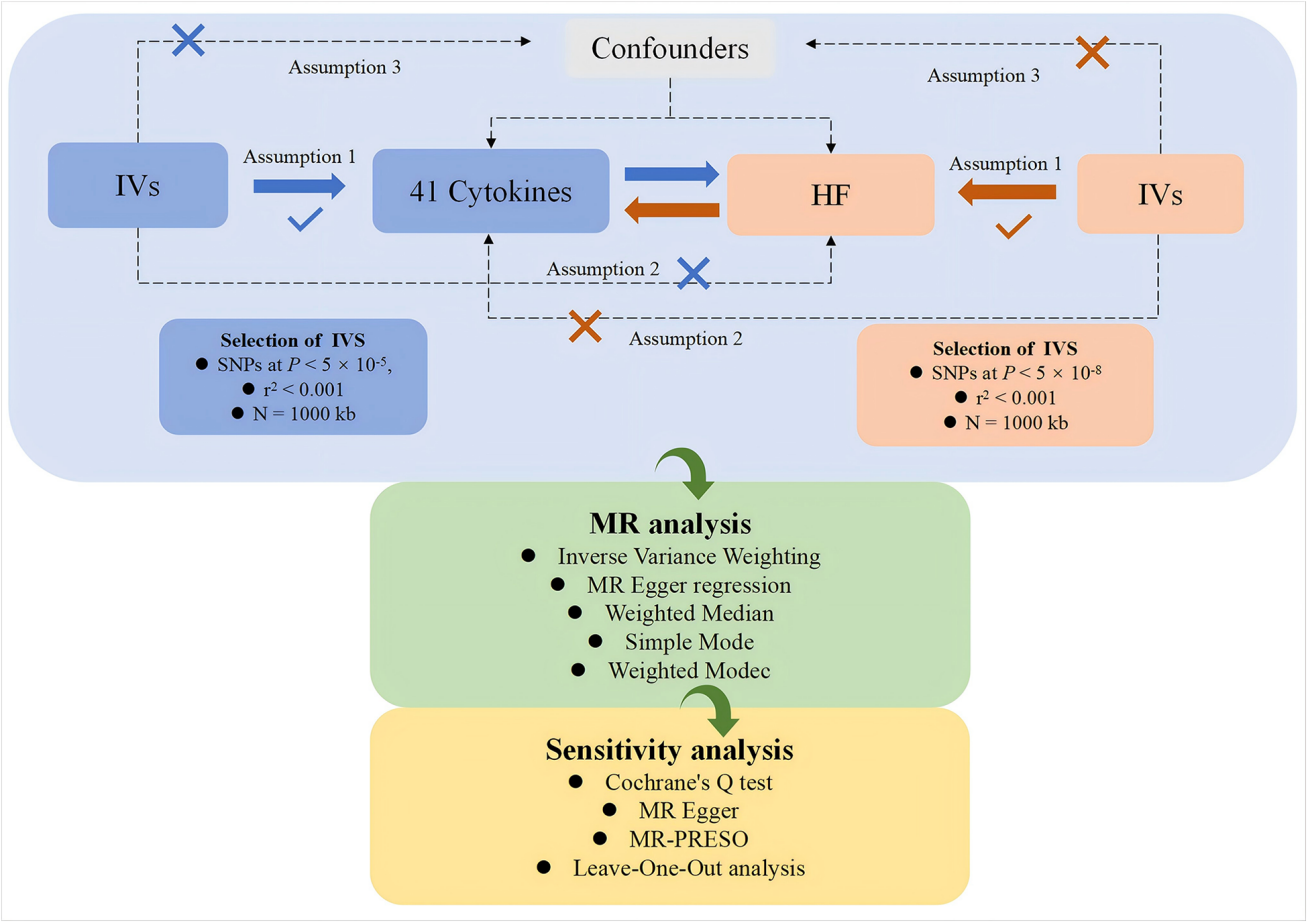
The study framework chart. IVs: instrumental variables; HF: heart failure.

This study, based on a genome-wide association study (GWAS) dataset, employs a two-sample, bidirectional MR approach to systematically investigate the potential causal relationships between various cytokines and HF. The study is conducted in two parts. In the first part, cytokines are considered as exposures, and genetic variations are used as IVs to investigate their relationship, with HF as the outcome. In the second part, the exposure and outcome are reversed for analysis. The flowchart of the study is shown in Figure 1. Both parts of the study adhere to the three core assumptions of MR analysis (22):1. Relevance (23): genetic variations are related to the exposures. 2. Independence (24): no confounding factors are involved in the exposure-outcome pathway. 3. Exclusivity (23): the genetic variation does not affect the outcome except through its association with the exposure.

### 2.2 Ethical considerations

Our study utilized publicly available GWAS datasets for MR analysis. Information regarding ethical committee approvals and participant informed consent can be found in the original research studies from each data source.

### 2.3 Data sources

To mitigate potential differences in the distribution of genetic variations across different ethnic populations, we used the largest and most recent Genome-Wide Association Study (GWAS) in European populations as the data source. The definition of the European population is that at least 80% of the participants have European ancestry. We utilized the HF GWAS dataset provided by the Heart Failure Molecular Epidemiology for Therapeutic Targets (HERMES), which is a meta-analysis involving 26 studies. Diagnoses were based on criteria including the 9th/10th revision of the International Classification of Diseases codes or physician diagnoses. Adjustments for age and sex were made in the analysis of SNPs. The dataset comprises a total of 47,309 HF patients and 930,014 controls, with a total of 7,773,021 SNPs (25). Diagnostic information and baseline characteristics for each cohort are provided in Supplementary Table S1.

The cytokine GWAS data is derived from a meta-analysis of circulating cytokine levels in the Finnish population, which includes 8,293 samples from 3 cohorts: the Cardiovascular Risk in Young Finns Study (YFS), a multi-center follow-up study; FINRISK, a cross-sectional study monitoring chronic disease risk factors in the Finnish population, conducted every five years, our study extracts the results of FINRISK's 1997 and 2002 cross-sectional surveys. Final sample sizes for the three cohorts were 1980, 1,705, and 4,608 respectively. Cytokine levels were measured in participants' fasting plasma, half-fasting EDTA plasma, and half-fasting heparin plasma. For more details on cytokine classification and information, please refer to the [(Supplementary Table S2) and Baseline (Supplementary Table S3)] (26).

### 2.4 Instrumental variables

In accordance with the three fundamental assumptions of MR (22), IVs must satisfy the following three conditions:1. IVs are significantly associated with the exposure factor: In this study, to obtain enough SNPs as IVs (27, 28), we selected SNPs with a genome-wide significance level of *P* < 5 × 10^−5^ and an *r*^2^ < 0.001, withisn a genomic locus width of *N* = 1,000 kb. This ensures that the selected SNPs are in linkage equilibrium, guaranteeing their independence from each other. 2. IVs are unrelated to the outcome factor: SNPs should have a significance level of *P* > 5 × 10^−8^ in their association with the outcome. 3. Exposure factors are unrelated to confounding factors. Additionally, we computed the F-statistic for each IV, F = beta^2^/se^2^ (29–31), with beta representing the SNP-exposure effect estimate and se denoting its standard error. IVs with F-statistics less than 10 were classified as “weak IVs” (24) and were excluded from the MR analysis for the reliability of the analysis.

### 2.5 Mendelian randomization analysis

With five MR methods from the TwoSampleMR package, the ratios between the effects of SNPs on exposure and on outcome were calculated, then the results of each SNP calculation were combined to assess the potential causal relationship between exposure and outcome. The five MR methods include Inverse Variance Weighting (IVW), MR Egger regression, Weighted Median method, Simple Mode, and Weighted Mode (17). Among these methods,IVW is considered the most efficient, it could provide unbiased estimates with maximum statistical power after controlling for pleiotropy (21). Therefore, the results obtained from IVW analysis serve as the primary reference, with statistical significance denoted by *P* < 0.05. The Weighted Median method is suitable for cases with a higher proportion of invalid IVs and can generate effect estimates even when a substantial portion, possibly up to 50%, of IVs are invalid (18). The MR-Egger method exhibits higher tolerance for SNPs with horizontal pleiotropy, the MR Egger does not force the regression line to pass through the origin, allowing for targeted gene pleiotropy in the included IVs. The MR-PRESSO method allows for the exclusion of specific SNPs by excluding outliers to obtain estimates that are closer to the true values (32).

### 2.6 Sensitivity analysis

If the MR analysis results of the five methods are consistent, it is considered a stable and reliable result. To assess whether there was heterogeneity among the selected IVs, Cochrane's *Q* test was first performed, and if there was no significant heterogeneity at *P* > 0.05, the Wald estimates for each SNP were analyzed using the IVW fixed-effects model; otherwise, the random-effects model was used. MR-Egger regression assesses the horizontal pleiotropy of the analysis by the size of the intercept; and in the scatterplot, the correlation between the intercept and 0 is tested; if *P* > 0.05, it means that there is no horizontal pleiotropy, and vice versa (32). In addition, select exposure factors with SNPs ≥ 4 for MR-PRESO analysis, set K = 10,000, eliminate outlier SNPs, and estimate the corrected results (18). Finally, assess the bias of individual SNPs in MR by Leave-One-Out sensitivity analysis, exclude SNPs that potentially influence the results one by one; then re-run MR analysis to ensure the stability of the results.

## 3 Results

### 3.1 Genetic instruments

In accordance with the filtering criteria mentioned earlier, relevant SNPs information was extracted, and the information for the SNPs used as IVs for exposures in the MR analysis can be found in Supplementary Tables S3 and S4, including the calculation of F-statistics. In the dataset for HF as the exposure, there are a total of 168 independent SNPs, and the F-statistics for individual SNPs range from 16.4398 to 83.0960. In the summary dataset for 41 cytokines, there are a total of 449 SNPs, with each cytokine having at least 3 SNPs. The F-statistics for individual cytokines range from 20.7962 to 782.4524, all of which are above 10. This suggests that, even after SNPs conditional cleaning in the HF and cytokine datasets, the genetic instruments remain robust and strong.

### 3.2 Impact of circulating cytokines on HF

**Figure 2:**
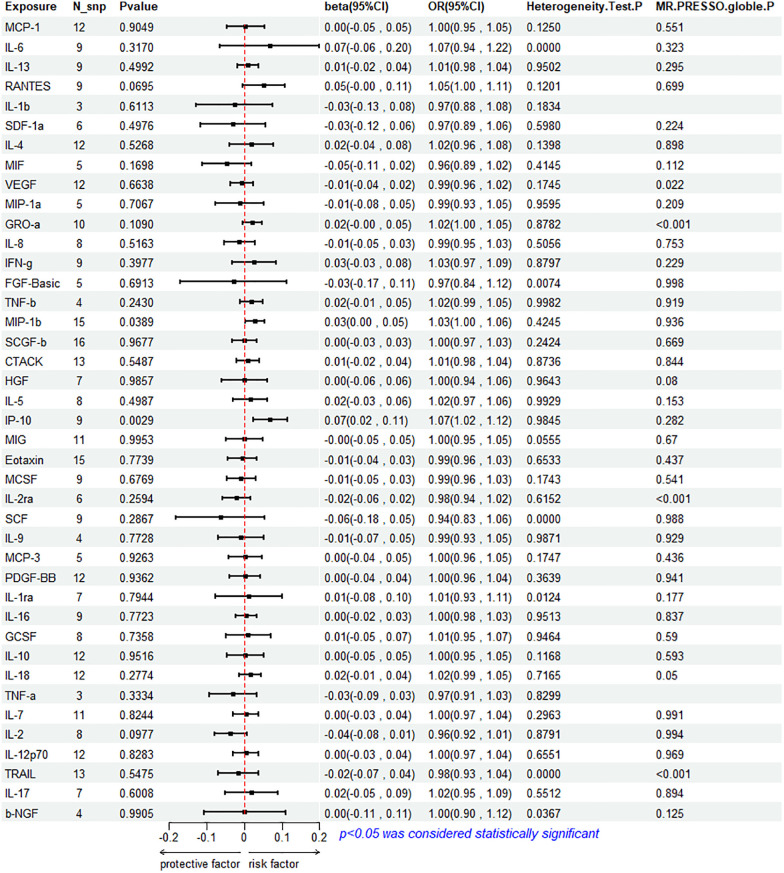
Mendelian randomization analysis of 41 cytokines and heart failure.

**Figure 3:**
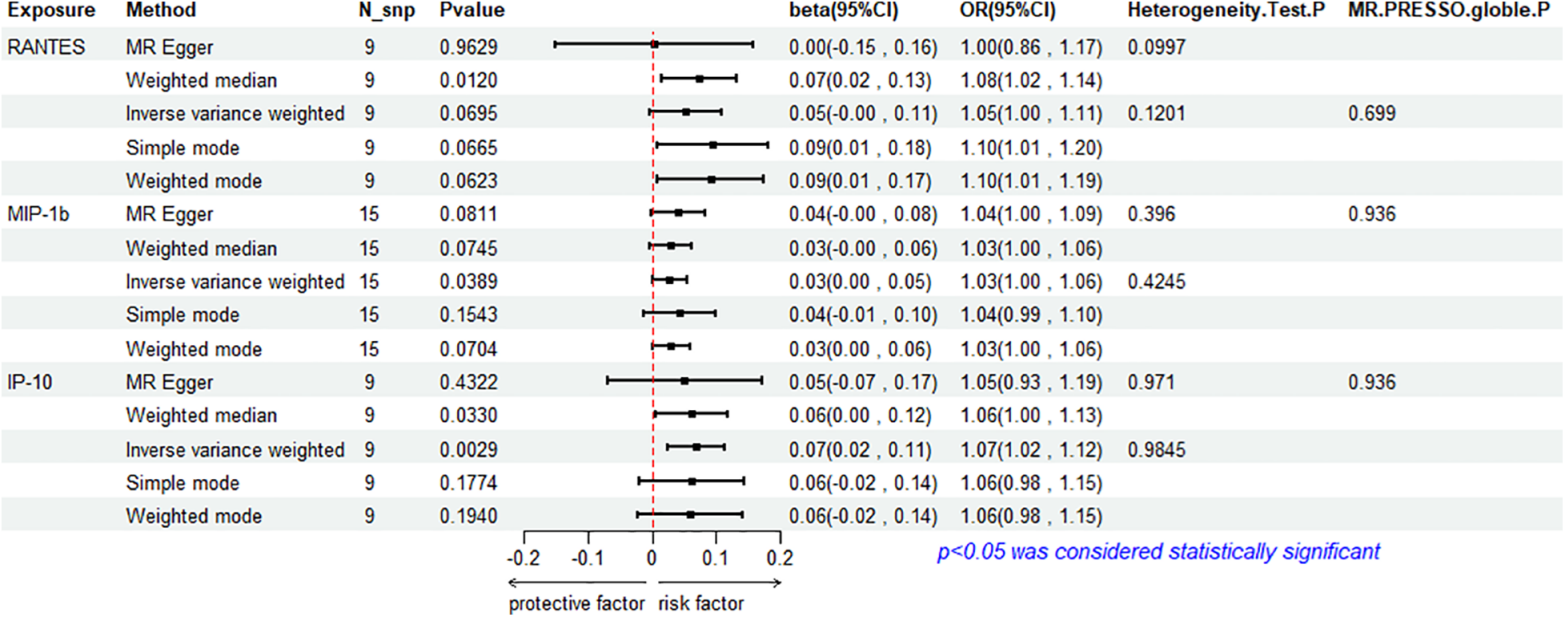
Mendelian randomization analysis of 3 cytokines and heart failure.

The results of the IVW method analysis for the impact of 41 cytokines on HF are presented in Figure 2. It shows that only two cytokines (MIP-1β and IP-10) exhibit a significant risk association with HF. Furthermore, RANTES demonstrates a stronger risk association with HF in the Weighted Median analysis, as detailed in Figure 3. For a comprehensive summary of the results of the 41 cytokines' impact on HF, refer to Supplementary Table S5.

#### 3.2.1 Association between MIP-1β and HF

High genetic prediction of circulating MIP-1β is associated with an increased risk of HF (beta = 0.278, beta 95% CI = 0.0014–0.0541, OR = 1.0282, OR 95% CI = 1.0014–1.0056, *P* = 0.0389). Cochrane's *Q* test (*P* = 0.4245) did not reveal any heterogeneity (Supplementary Table S6). A fixed-effects model was used for the IVW method. All five MR analysis methods consistently show a positive association. The MR-Egger regression intercept is close to 0 (MR Egger-intercept = −0.0048, MR Egger-intercept *P* = 0.4455), suggesting no directional pleiotropy. The funnel plot is relatively symmetrical on both sides. Leave-one-out sensitivity analysis did not yield different results (Supplementary Figure S1). MR-PRESSO also found no evidence of horizontal pleiotropy (global *P* = 0.936), indicating the reliability of the MR analysis.

#### 3.2.2 Association between IP-10 and HF

The IVW method reveals that an increase in plasma IP-10 levels is also associated with an increased risk of HF (beta = 0.0689, beta 95% CI = 0.0236–0.1141, OR = 1.0713, OR 95% CI = 1.0239–1.1209, *P* = 0.0029). All five MR methods show consistent risk relationships. Cochrane's *Q* test (*P* = 0.9845) did not reveal heterogeneity (Supplementary Table S6). The IVW method was conducted using a fixed-effects model. The MR-Egger regression intercept (MR Egger-intercept = 0.0032, MR Egger-intercept *P* = 0.7636) indicates no significant directional pleiotropy. MR-PRESSO testing yielded a global *P*-value of 0.282. Although the funnel plot is asymmetrical on both sides, leave-one-out sensitivity analysis did not produce different results (Supplementary Figure S1).

#### 3.2.3 Association between RANTES and HF

RANTES, on the other hand, demonstrates a stronger risk association with HF in the Weighted Median analysis (beta = 0.00738, beta 95% CI = 0.0162–0.1313, OR = 1.0765, OR 95% CI = 1.0164–1.1403, *P* = 0.0120). All five MR analysis results show consistent risk associations. The IVW method used a random-effects model, and the IVW analysis results remain non-significant (*P* = 0.0695) (Supplementary Table S5). The Cochrane's *Q* test (*P* = 0.1201) also did not reveal heterogeneity (Supplementary Table S6). MR-Egger regression intercept is close to 0 (MR Egger-intercept = 0.0097, MR Egger-intercept *P* = 0.5320), indicating no significant directional pleiotropy. MR-PRESSO testing found no significant horizontal pleiotropy (global *P* = 0.699). The funnel plot for the IVW method is relatively symmetric on both sides. Leave-one-out sensitivity analysis did not yield significantly different results (Supplementary Figure S1).

### 3.3 Impact of HF on circulating cytokine

**Figure 4:**
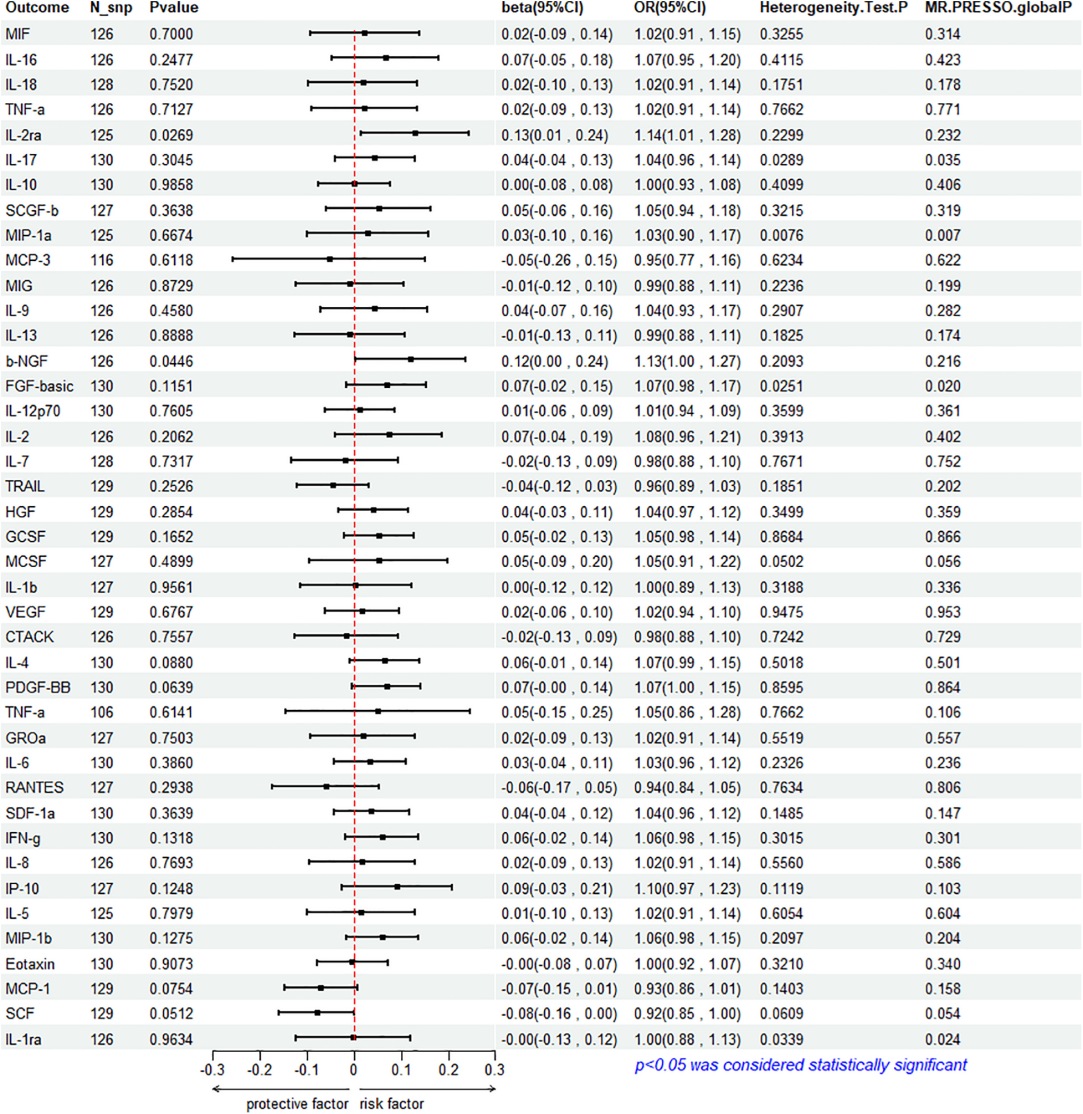
Mendelian randomization analysis of heart failure and 41 inflammatory factors.

**Figure 5:**
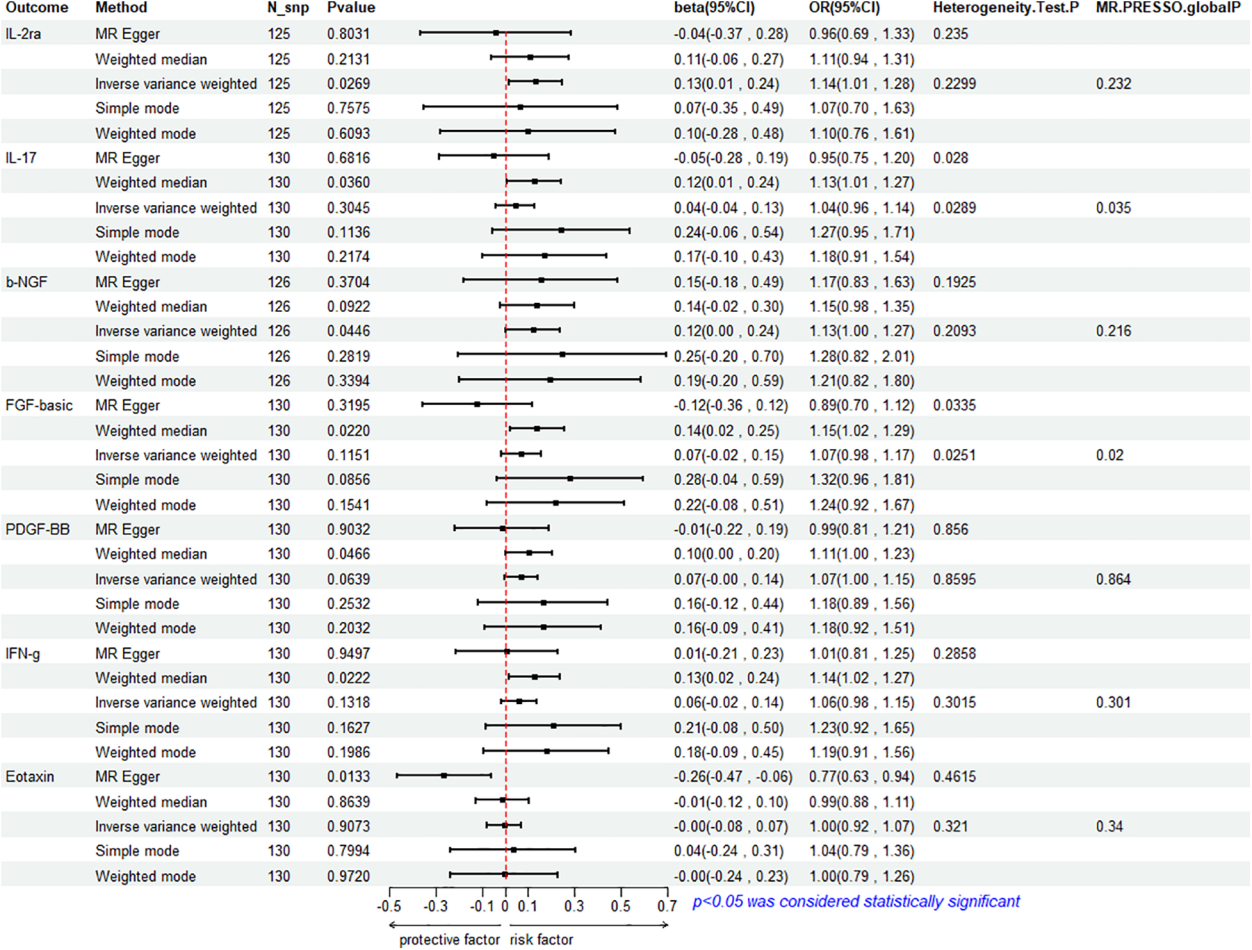
Mendelian analysis of heart failure and 7 cytokines.

To assess the reverse effect, we used HF as the exposure factor and extracted seven cytokines that showed a significant correlation. The results of the IVW method analysis for the impact of HF on plasma cytokine levels are presented in Figure 4, which shows that the occurrence of HF affects the expression of two cytokines (IL-2ra and β-NGF). In addition, four cytokines (IL-17, FGF-basic, PDGF-BB, and IFN-*γ*) exhibit a stronger positive correlation in the Weighted Median analysis, while Eotaxin shows a significant negative correlation with HF in the MR Egger method. More details refer to Figure 5. (Supplementary Table S10).

#### 3.3.1 Association between HF and IL-2ra

The IVW method reveals a significant correlation between genetically predicted HF and increased circulating IL-2ra levels (beta = 0.1292, beta 95% CI = 0.0147–0.2436, OR = 1.1379, OR 95% CI = 1.0148–1.2759, *P* = 0.0296). Heterogeneity testing using Cochrane's *Q* test (*P* = 0.2299) did not reveal heterogeneity (Supplementary Table S11). The IVW method used a fixed-effects model. The funnel plot is generally symmetrical on both sides. Leave-one-out sensitivity analysis showed relatively robust results (Supplementary Figure S1). MR-PRESSO analysis did not detect any horizontal pleiotropy (global *P* = 0.232), indicating the robustness of the IVW results. When using the MR Egger method, beta = −0.0413, OR=0.9595, indicating a negative association with HF. Cochrane's *Q* test resulted in a *P*-value of 0.2299, and the MR-Egger regression intercept was close to 0 (MR Egger-intercept = 0.0089, MR Egger-intercept *P* = 0.2726), suggesting no significant directional pleiotropy.

#### 3.3.2 Association between HF and β-NGF

According to the IVW method, HF is closely related to an increase in circulating β-NGF levels (beta = 0.1203, beta 95% CI = 0.0029–0.2376, OR = 1.1272, OR 95% CI = 1.0029–1.2682, *P* = 0.0446). Heterogeneity testing using Cochrane's *Q* test (*P* = 0.2093) did not reveal heterogeneity (Supplementary Table S11). The IVW method used a fixed-effects model. The funnel plot is generally symmetrical on both sides. Leave-one-out sensitivity analysis showed relatively robust results. However, results from MR Egger, Weighted Median, Simple Mode, and Weighted Mode were consistent in direction but not statistically significant. The MR-Egger regression intercept was 0.0089, and the MR Egger-intercept *P*-value = 0.8379, suggesting no significant directional pleiotropy.

#### 3.3.3 Association between HF with IL-17, FGF basic, PDGF-BB and IFN- *γ*

The results of Weighted Media suggest that IL-17 (beta = 0.1242, beta 95% CI = 0.0081∼0.2402, OR = 1.1322, OR 95% CI = 1.0082–1.2715, *P* = 0.0360), FGF basic (beta = 0.1369, beta 95% CI = 0.0197–0.2541, OR = 1.1467, OR 95% CI = 1.0199–1.2893, *P* = 0.0220), PDGF-BB (beta = 0.1027, beta 95%CI = 0.0015–0.2039, OR = 1.1082, OR 95% CI = 1.0015–1.2261, *P* = 0.0466), and IFN- *γ* (beta = 0.1283, beta 95% CI = 0.0183–0.2384, OR = 1.1369, OR 95% CI = 1.0185–1.2692, *P* = 0.0222) are significantly associated with the risk of HF. FGF basic and IL-17 exhibit heterogeneity, with Cochrane's *Q* test *P* of 0.0280 and 0.0335, respectively. The funnel plot is asymmetric and biased towards one side. Although the intercept of the MR Egger pleiotropy test for the four cytokines was close to 0 and there was no direct significant pleiotropy, MR-PRESSO analysis indicated potential horizontal pleiotropy for FGF-basic (global *P* = 0.02) and IL-17 (global *P* = 0.035), suggesting possible bias. However, after removing outliers, heterogeneity still persisted, indicating that the correlation could not be obtained for FGF-basic and IL-17 in all methods except Weighted Median, which compensates for the credibility of the results. Furthermore, leave-one-out forest plots showed that the effect of HF on the levels of these four cytokines remained relatively stable.

#### 3.3.4 Association between HF and Eotaxin

Eotaxin shows a significant protective correlation with HF in the MR Egger method. HF is associated with a decrease in plasma Eotaxin levels (beta = −0.2632, beta 95% CI = −0.4685 to −0.0578, OR = 0.7686, OR 95% CI = 0.6259–0.9438, *P* = 0.0133). Cochrane's *Q* test (*P* = 0.4615) did not reveal heterogeneity (Supplementary Table S11). The IVW method used a fixed-effects model. While the Simple Mode method showed a positive correlation, the results from the other four MR methods were all negative. The funnel plot is generally symmetrical on both sides. The MR-Egger regression intercept was 0.0137, and the MR Egger-intercept *P*-value was 0.0092, suggesting no directional pleiotropy. MR-PRESSO also found no horizontal pleiotropy (global *P* = 0.34). Leave-one-out sensitivity analysis did not yield significantly different results, after removing SNP (rs660240, rs10846742, rs10152199, rs117734706, rs35054810, rs17496249, rs78957967, rs146379019, rs1757223, rs34592354, rs77932705), the results are relatively robust.

## 4 Discussion

In recent years, there has been extensive focus on the abnormal expression of biologically active factors in the development and progression of HF. Cytokines have received particular attention, following neurohormones, for their widespread mention in this context (33, 34). However, most of the previous studies of HF and cytokines have been observational, making it difficult to infer directed causation. There are also few studies of interventional cytokines. The natural randomized design of MR studies provides a higher level of clinical evidence. But the only MR studies that provide evidence at the gene level are unidirectional (15, 16), failed to identify inflammatory markers with a clear correlation with HF. The relationships between more cytokines, especially those displaying reverse effect, with HF are yet to be explored. To the best of our knowledge, this study is the first bidirectional, two-sample MR study of cytokines and HF, and utilizes the latest and largest genome-wide association data to evaluate the correlation between 41 cytokines and HF. Through this bidirectional analytical strategy, we could differentiate between upstream and downstream factors in the disease process, thus explore the pathophysiological mechanisms of HF. In the bidirectional MR analysis, we observed that elevated circulating levels of MIP-1β, IP-10, and RANTES, determined by genetics, were associated with an increased risk of HF. Conversely, the genetic susceptibility to HF was associated with elevated circulating levels of IL-2ra, β-NGF, IL-17, FGF-basic, PDGF-BB, IFN-*γ*, and a decrease in Eotaxin levels. Our study identified the responsible factors at various stages of HF, confirming the cytokine hypothesis in HF. Circulating cytokines play a crucial role in the formation and progression of HF, and HF may lead to worse outcomes through potential pro-inflammatory effects. Throughout the research, we conducted Cochrane's *Q*, leave-one-out heterogeneity tests, and MR-PRESSO and MR-Egger were applied for pleiotropy test to assess the reliability of MR analysis.

Previous results suggest that the elevated levels of MIP-1β are associated with an increased risk of HF. MIP-1β is a proinflammatory cytokine that can enhance the inflammatory response and promote inflammation progression. Observational studies (35) have found higher levels of MIP-1β in HF patients compared to the healthy group. Genetic testing (36) revealed that the mRNA expression of MIP-1β, which is regulated in HF patients, is eight times higher than that in the healthy control group. Furthermore, the corresponding receptor gene, CCR2, was also 2-fold higher than that of the control group and was negatively correlated with LVEF. IP-10 is a chemokine secreted by various cells, such as T cells, neutrophils, and endothelial cells (37), and it can recruit T cells, promote T cell differentiation, and regulate angiogenesis by binding to the chemokine receptor CXCR3 (38). We found that IP-10 is positively associated with the occurrence of HF, which aligns with previous unidirectional MR studies (39). In a mouse model of diastolic left ventricular dysfunction, the average increase in plasma IP-10 concentration is 140% of the NT-proBNP levels (40). The MONICA/KORA Augsburg 1984–2002 cohort study (41) discovered a positive association between IP-10 and several HF risk factors, including age, BMI, and coronary artery disease, and elevated plasma IP-10 levels precede the occurrence of coronary artery disease. Results from two prospective cohort studies also indicate that higher IP-10 concentrations are associated with an increased risk of HF (42). In our study, RANTES exhibited a strong positive association with HF in the Weighted Median analysis. RANTES, also known as CCL5, is primarily secreted by normal T cells and is involved in the transportation, homing, and proliferation of T cells (39). A decrease in serum RANTES levels with the severity of myocardial infarction was observed in patients with post-myocardial infarction HF (42), which contradicts our findings. The possible reasons for this discrepancy are mainly: first, the genetic information from genes to proteins has to go through the process of transcription and translation, and this process is regulated by many factors, so the discrepancy between the genetic information and the phenotype may occur; second, the level of RANTES in circulation could be influenced by different stages of the neuro-hormonal-immune pathway, so it may have pleiotropic effects at different stages of HF (43), leading to inconsistent observations. However, these hypotheses need to be verified by more animal experiments and clinical trials.

In this study from HF to circulating cytokines, we observed a significant increase in plasma IL-2ra levels with the occurrence of HF. IL-2ra is one of the heterotrimers that make up the high-affinity IL-2 receptor (IL-2r), which is involved in T cell activation and immune responses (44). Previous research has found increased serum IL-2R levels in patients with stable angina (45) and coronary artery disease (46). A case-control study at the genetic level (47) identified a significant association between a single SNP (rs12569923) in IL-2ra and the increased risk of diabetes in coronary artery disease patients. β-NGF is a neurotrophic factor involved in the differentiation of central and peripheral neurons and selective innervation of target tissues (48). NGF affects the strength and quantity of the development of synapses, enhances synaptic transmission between autonomic neurons and cardiomyocyte (49); in addition, overexpressed NGF binds to its high-affinity transmembrane receptor TrKA, leading to its dimerization and activation, which recruits and activates downstream signaling molecules through a signaling cascade reaction, triggering a variety of physiological responses (50), improving endothelial cell survival and promoting neovascularization (51). It was shown that NGF can support the survival of sympathetic neurons at low levels and synaptic transmission requires over ten times of the normal NGF levels (49). In patients with HF and in rats with post-myocardial infarction HF, NGF expression decreases due to the dysfunction of cardiac sympathetic nerve endings (52). However, in mice with acute myocardial infarction, delivering NGF-related genes around the infarcted myocardium can improve myocardial cell survival and cardiac function (51), suggesting that NGF can participate in cardiac repair and therapy. IL-17 is an interleukin, and consistent with MR results, studies have found increased plasma IL-17 levels in patients with congestive HF (53). In a study on calcium in HF patients (54), IL-17 levels in the circulation and cardiac tissue were significantly increased in HF patients and mouse models. Knockdown of IL-17-related genes in mice increased the magnitude of calcium transients and cell shortening, ultimately improving heart function. In patients with ischemic HF, anti-IL-17 neutralizing antibodies can inhibit myocardial cell fibrosis and apoptosis, providing cardiac protection (55).

IFN-*γ* is a proinflammatory cytokine with antiviral, antitumor, and immune-regulatory functions. Animal experiments have shown an increase in plasma IFN-*γ* levels in mice with post-myocardial infarction HF (56), consistent with our MR results. Interestingly, a recent clinical observational study found lower IFN-*γ* levels in HF patients compared to healthy patients, which aligns with the IVW results. However, this negative correlation trend did not extend to the correlation between disease duration and LVEF. Instead, IL-9 levels, which did not exhibit significant correlation in our MR results, were negatively correlated with LVEF and duration of illness (35). Eotaxin is a key chemokine in the inflammatory process and is involved in the recruitment of eosinophils at the site of injury. MR results revealed a gradual decrease in plasma Eotaxin levels with the onset of HF, which may be related to the rarity of eosinophils in atherosclerotic lesions (57). However, Eotaxin and its receptor CCR3 were found to be elevated in both atherosclerotic and healthy control populations (58). A cohort study found that a T/A mutation in amino acid 23 on the Eotaxin gene increased the risk of myocardial infarction (57), and provided evidence for the involvement of Eotaxin in the formation of atherosclerosis, also providing evidence for the involvement of Eotaxin in the formation of atherosclerosis.

Our findings emphasize the intricate relationship between cytokines and HF. Interestingly, there was no overlap between cytokines associated with increased risk of HF and those expressed as a result of HF. This suggests that the mechanisms underlying the onset and progression of HF may be different. Cohort studies have demonstrated a dose-dependent relationship between higher than baseline body mass index (BMI) levels and downstream risk of HF hospitalization events (59). In HF patients, the adverse outcomes were dominated by renal failure, infection, and multi-organ failure, while the prevalence of chronic kidney disease in HF patients was 63%, with an 11% increase in hospitalization and a 17% increase in mortality; meanwhile, there was a U-shaped relationship between eGFR and mortality in HF patients (60). Given these conflicting findings, further research, particularly focusing on underlying mechanisms, is essential to clarify the potential role of cytokines in the development of HF. Its mechanism may be related to increased oxidative stress, chronic inflammation, myocardial hypertrophy, perivascular and interstitial collagen deposition/cross-linking, abnormal intracellular calcium processing, endothelial and mitochondrial dysfunction, and cell apoptosis (61).

Cytokine therapy was one of the earliest forms of immunotherapy approved by the US Food and Drug Administration (FDA). Research has found that in patients with non ST segment elevation myocardial infarction, administering a single dose of tocilizumab before coronary angiography can reduce the release of troponin T and systemic inflammation (62). The efficacy of TNF-α antagonism in patients with HF has been further investigated through the administration of soluble TNFR, but no significant clinical benefit has been observed (63). To date, the clinical benefit of cytokine therapy has not surpassed that of current classic agents such as diuretics. Moreover, the utilization of cytokine therapy may impact the inflammatory state in patients with co-infection. The heterogeneity of HF patients in terms of disease complexity and severity poses challenges for cytokine-based therapy implementation. Long-term administration of cytokine therapy is deemed suboptimal. HF may not be an ideal candidate disease for cytokine therapy due to its complex nature. But that doesn't mean it's not worth pursuing, brief cytokine therapy in conditions such as myocardial infarction and myocarditis has shown potential in altering inflammatory responses and potentially preventing progression towards HF. Of all these cytokines, MIP-1β, IP-10, RANTES, IL-2r, β-NGF, PDGF-BB, FGF-basic, IL-17, and IFN-*γ* are especially desirable for immediate study.

The novelty of this study lies in the fact that it is the first time that a bidirectional, two-sample MR approach was employed to explore the association between circulating cytokines and HF. The database of this study is the largest GWAS dataset on circulating cytokines and HF ever published. Due to the natural randomization of the MR and the strict screening criteria for SNPs, the results of the study are less susceptible to confounding factors and reverse causal associations. However, our study also has some limitations. First, the data sources for our exposure and outcome are based on studies conducted on populations of European ancestry, and the exposures and outcomes did not originate from the same population, so it is unclear whether these findings are applicable to other ethnicities or regions. Second, the relationship between cytokines and different phenotypes of HF still needs to be further explored because of the lack of datasets stratified by cardiac function class and ejection fraction. Third, to assess the relationship between as many cytokines as possible and HF, we used SNPs for exposure factors with a significance of *P* < 5 × 10^−5^, and the process of IVs screening may still carry some risk of confounders, such as the possibility that different cytokines may be confounders for each other. Finally, the dataset of circulating cytokines we used did not provide effector allele frequencies (EAFs), and there may be differences in the direction of alignment of the SNPs for exposure and outcome, but we also applied MR-Egger and MR-PRESSO tests for horizontal pleiotropy to reduce bias. In the future MR studies, in order to minimize all these bias, larger gene-wide databases of cytokines and diseases should be established, which should contain multi-ethnic populations and disease phenotypes. And animal experiments and clinical trials should also be used to validate the results of MR analyses and to provide more scientific evidence for the conclusions.

In conclusion, our study indicates that, among people of European ancestry, genetically increased plasma levels of MIP-1β, IP-10, and RANTES are positively associated with an increased risk of HF, while genetically increased risk of HF is associated with elevated plasma levels of IL-17, FGF-basic, PDGF-BB, IFN-*γ*, and decreased levels of Eotaxin. These findings provide an epidemiologic basis for cytokine-targeted drugs to prevent and treat HF, however, more animal experiments, clinical trials based on large samples is still needed in the future to support the conclusions at the gene level.

## Data Availability

The original contributions presented in the study are included in the article/Supplementary material, further inquiries can be directed to the corresponding authors.
