# Supplementary material for: The role of circulating cytokines in heart failure: a bidirectional, two-sample Mendelian randomization study
Source: Front Cardiovasc Med. 2024 Oct 22;11:1332015. doi: 10.3389/fcvm.2024.1332015 (PMC11534875; doi:10.3389/fcvm.2024.1332015)
Supplement: Supplementary file 2 [file Datasheet1.pdf]

Supplementary material

Cohort descriptions

Heart Failure Molecular Epidemiology for Therapeutic Targets

A meta-analysis of 26 studies, including 930,014 controls and 47,309 HF cases, conducted by the Heart Failure Molecular Epidemiology for Therapeutic Targets (HERMES) (https://www.hermesconsortium.org/) consortium revealed 12 independent variants linked to HF in 11 genomic regions.

The Cardiovascular Risk in Young Finns Study

The Cardiovascular Risk in Young Finns Study (YFS) is a multicentre follow-up study with randomly chosen subjects from the Finnish cities of Helsinki, Kuopio, Oulu, Tampere, and Turku and their rural surroundings. The study began in 1980 when 3,596 children and young adults participated in the first cross-sectional survey. The follow-up visits have been conducted in 1983, 1986, 1989, 2001, 2007, and 2011. The present cross-sectional study includes 1980 unrelated individuals who participated in the 2007 follow-up and who had both cytokine measurements and genotype data available. All participants gave written informed consent and the study was approved by local ethics committees1 .

FINRISK

FINRISK surveys are population-based cross-sectional studies conducted every 5 years to monitor the levels of chronic disease risk factors in Finland. Each survey includes 25- to 74-year-old randomly chosen subjects from five geographical areas of Finland. The present study analyses cytokine data from participants of the 1997 and 2002 surveys. In FINRISK 2002 the cytokine panel was run for a subset of participants that were older than 51 years. The study visit includes a clinical examination and semi-fasting blood sampling. The study was approved by the Coordinating Ethics Committee of the Helsinki and Uusimaa Hospital District and all study participants gave written informed consent.

Figure S1 Scatter plot, funnel plot, and forest plot of SNPs associated with IP-10, MIP-1β, RANTES and their risk of Heart Failure.

A IP-10

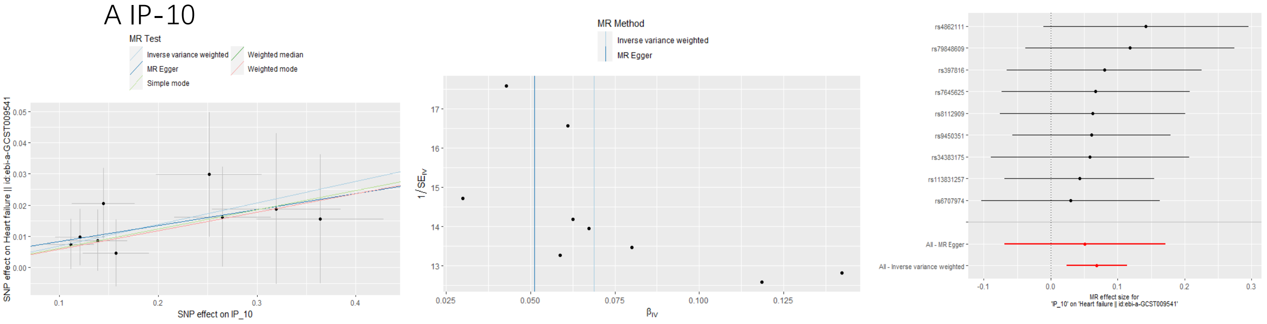

B MIP-1β

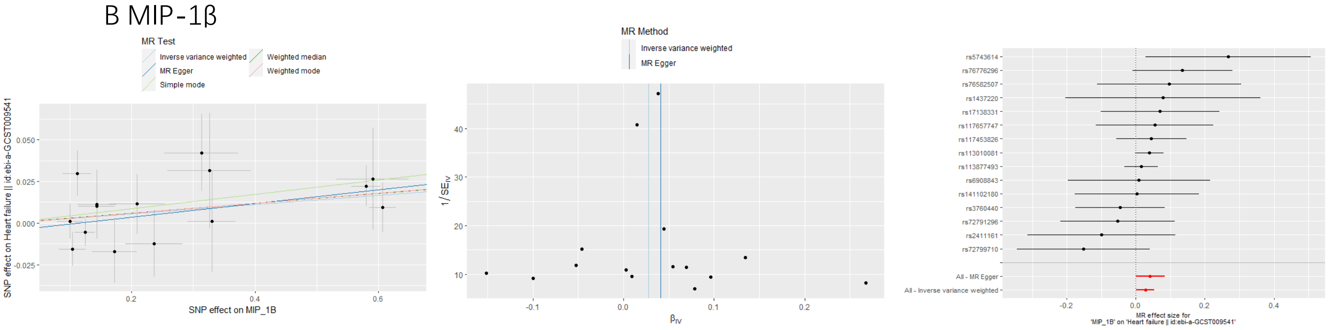

C RANTES

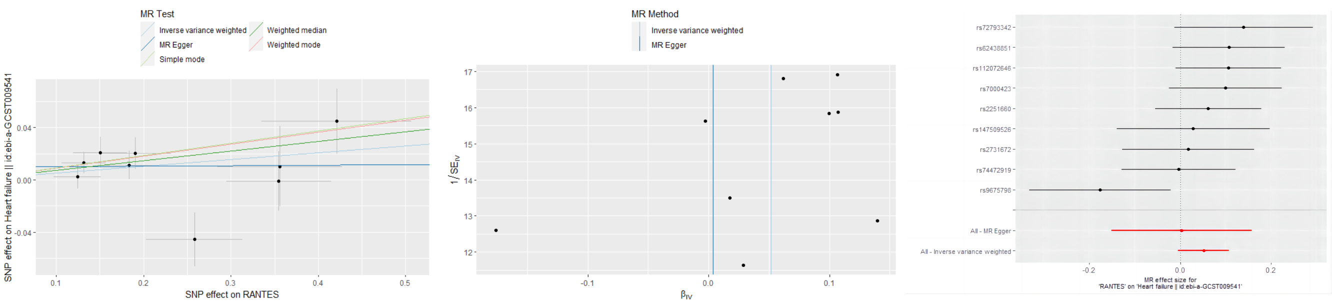

Figure S2 Scatter plot, funnel plot, and forest plot of SNPs associated with Heart Failure and their risk of β-NGF, EOTAXIN, FGF-basic, IFN-γ, IL-2ra, IL-17 and PDGF-BB.

A β-NGF

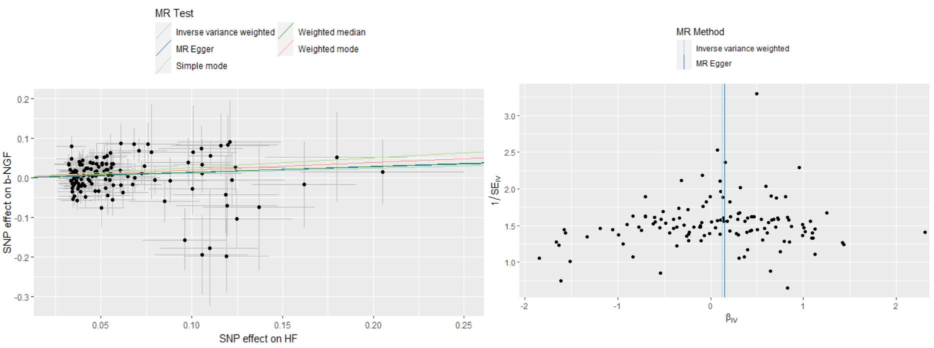

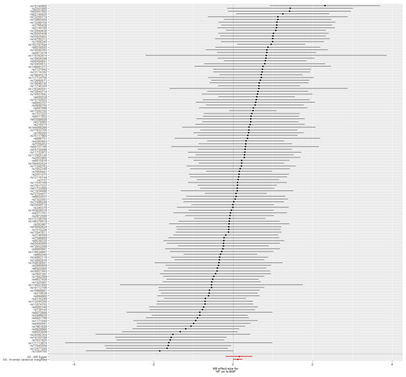

B Eotaxin

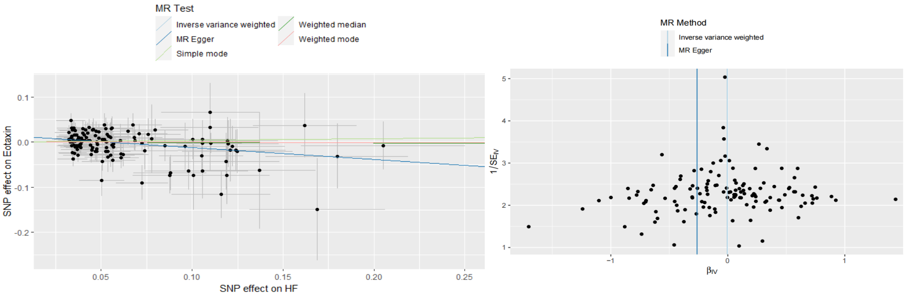

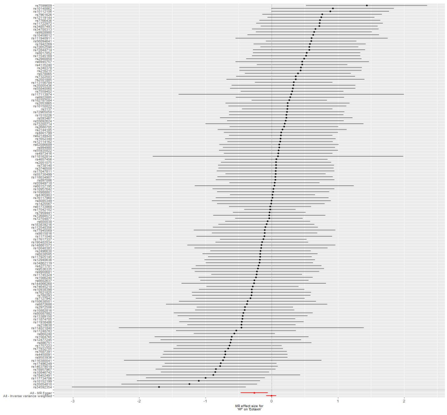

C FGF-basic

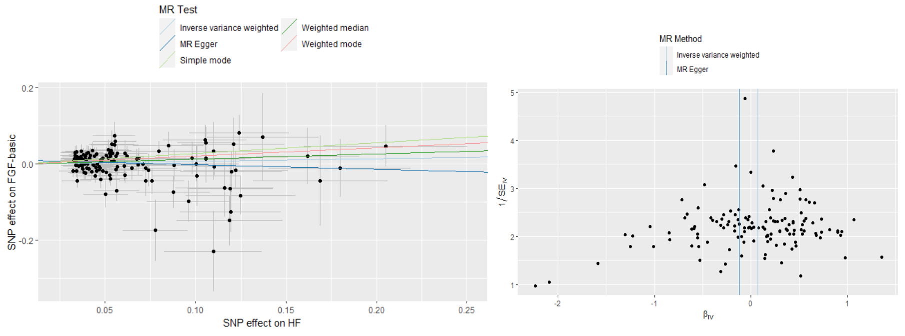

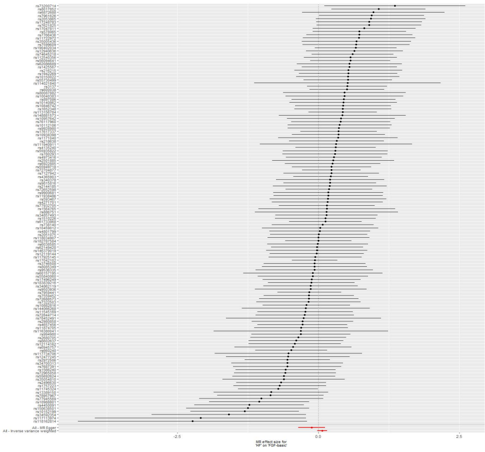

D IFN-γ

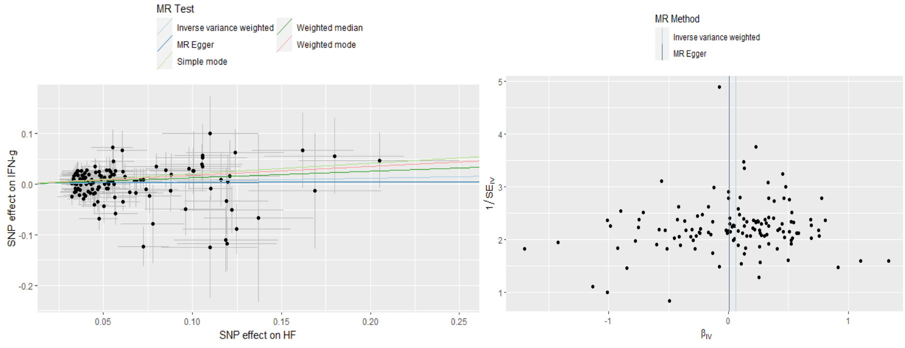

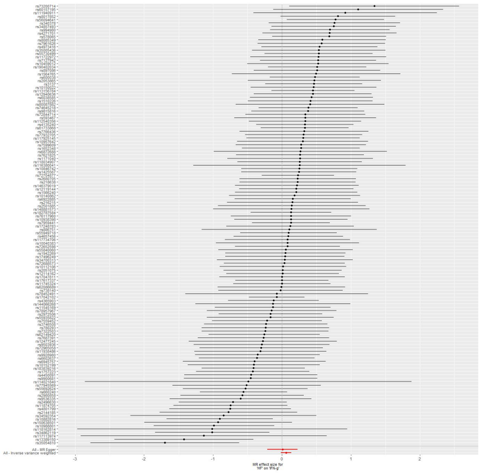

E IL-2ra

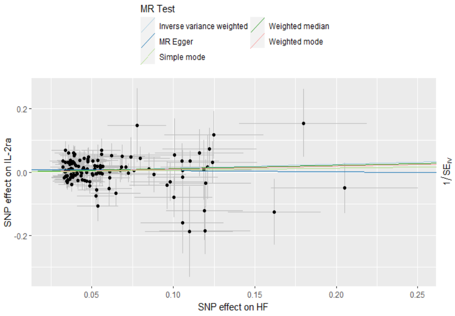

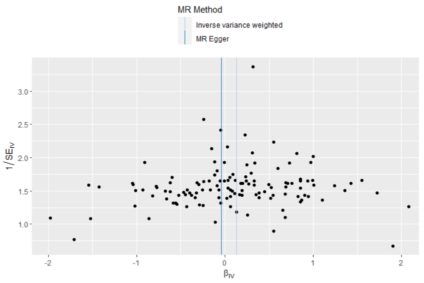

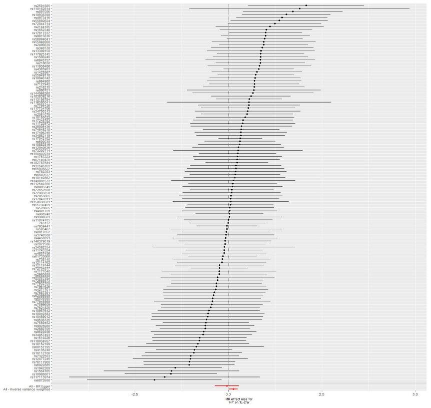

F IL-17

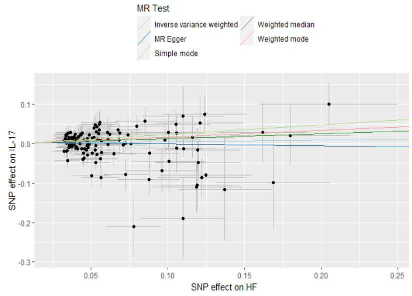

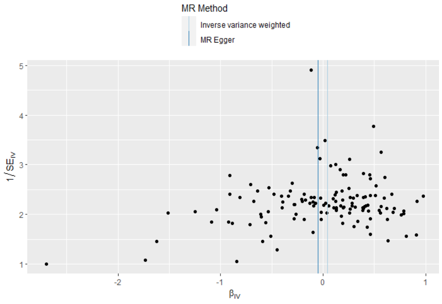

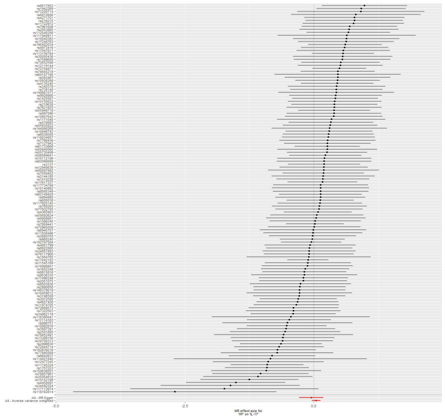

G PDGF-BB

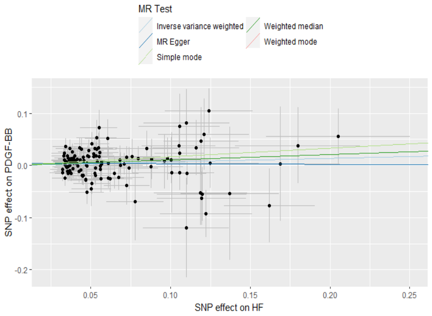

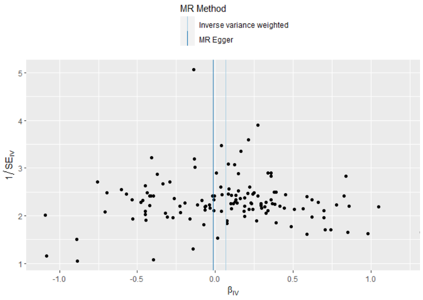

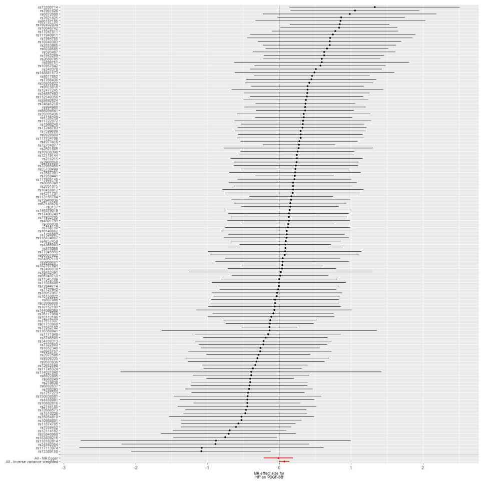

Figure S3 Effects of IP-10, MIP-1β, and RANTES on Heart Failure by the Leave-One-Out Method.

A IP-10

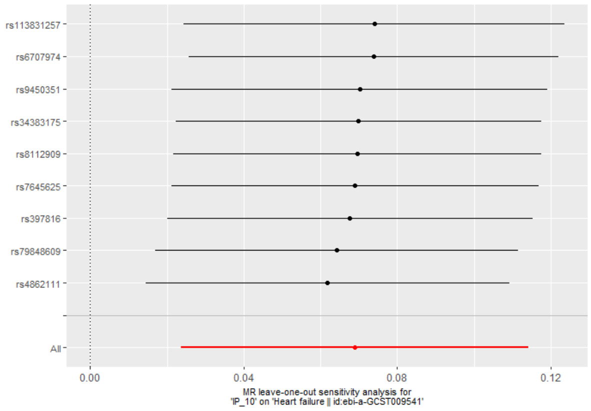

B MIP-1β

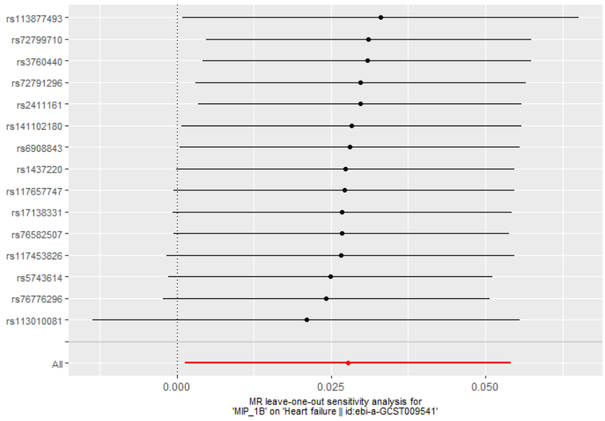

C RANTES

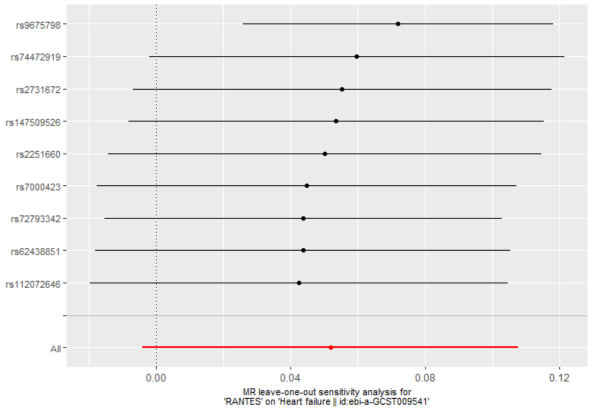

Figure S4 Effects of Heart Failure on β-NGF, EOTAXIN, FGF-basic, IFN-γ, IL-2ra, IL-17 and PDGF-BB by the Leave-One-Out Method.

A β-NGF

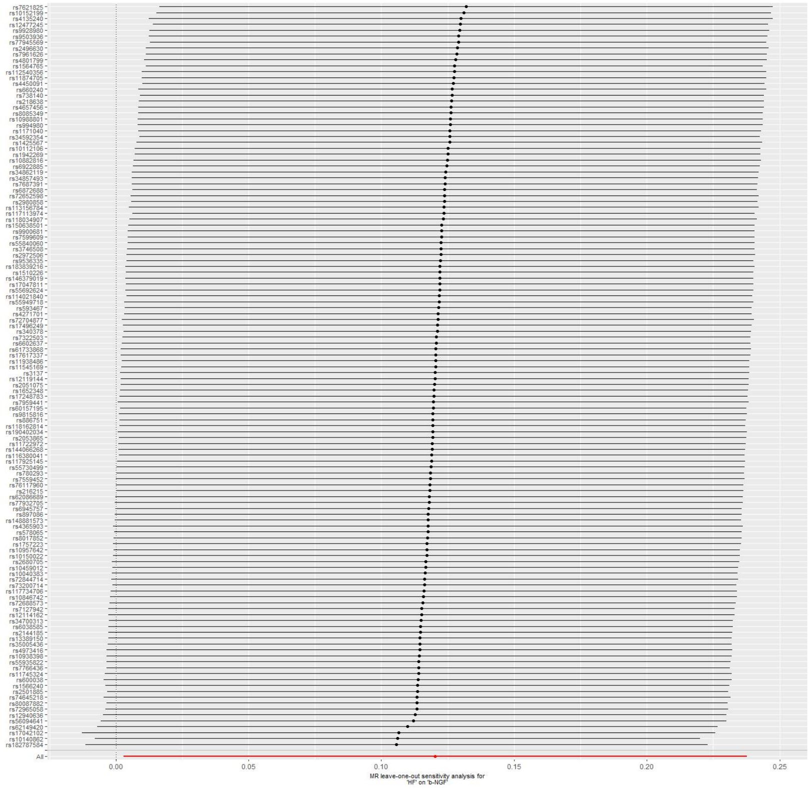

B Eotaxin

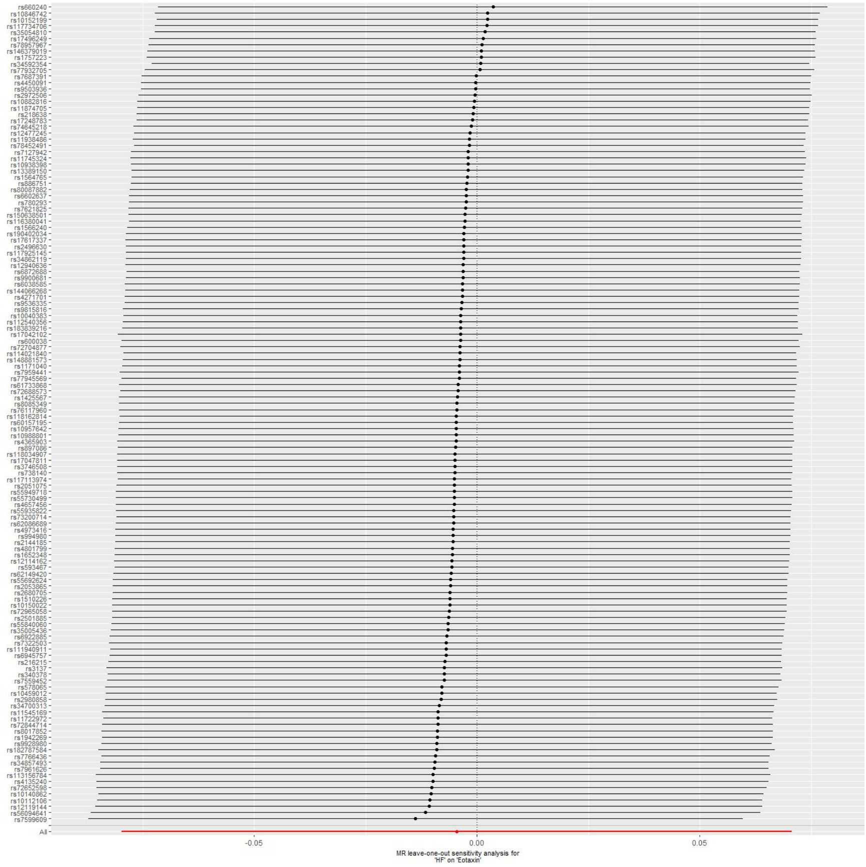

C IFN-γ

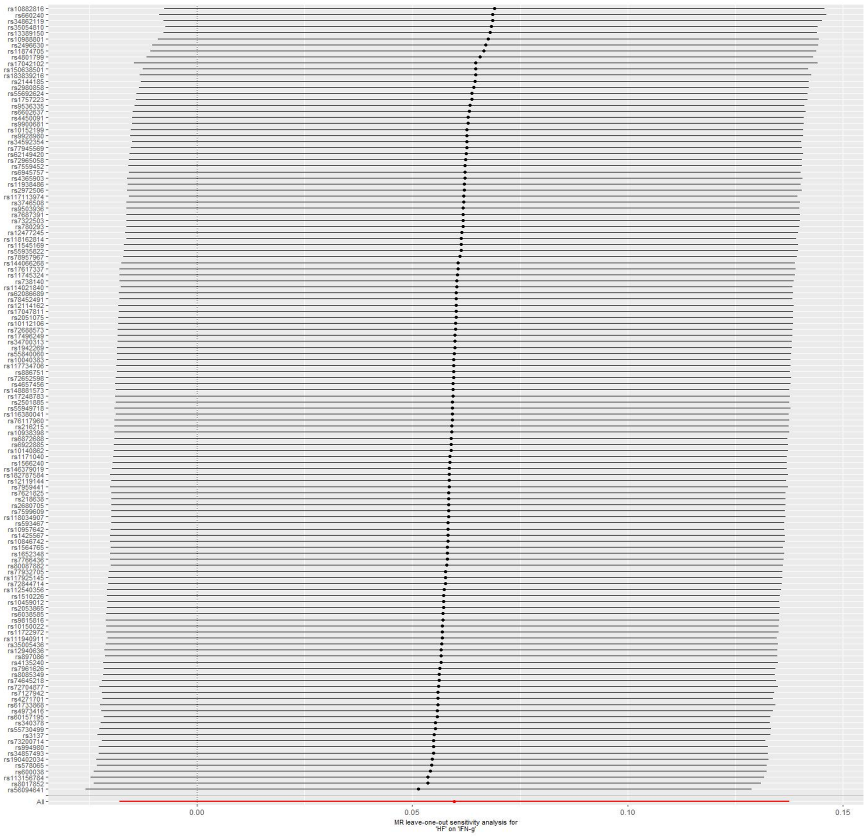

D IL-2ra

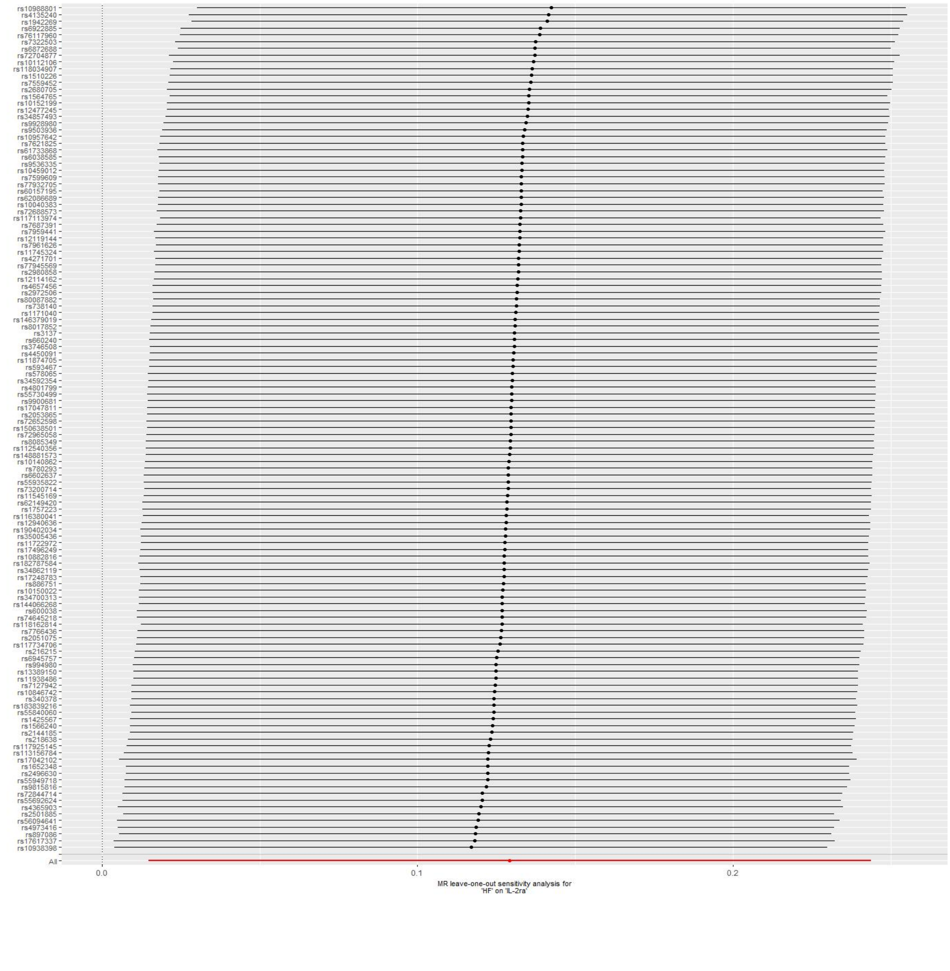

E IL-17

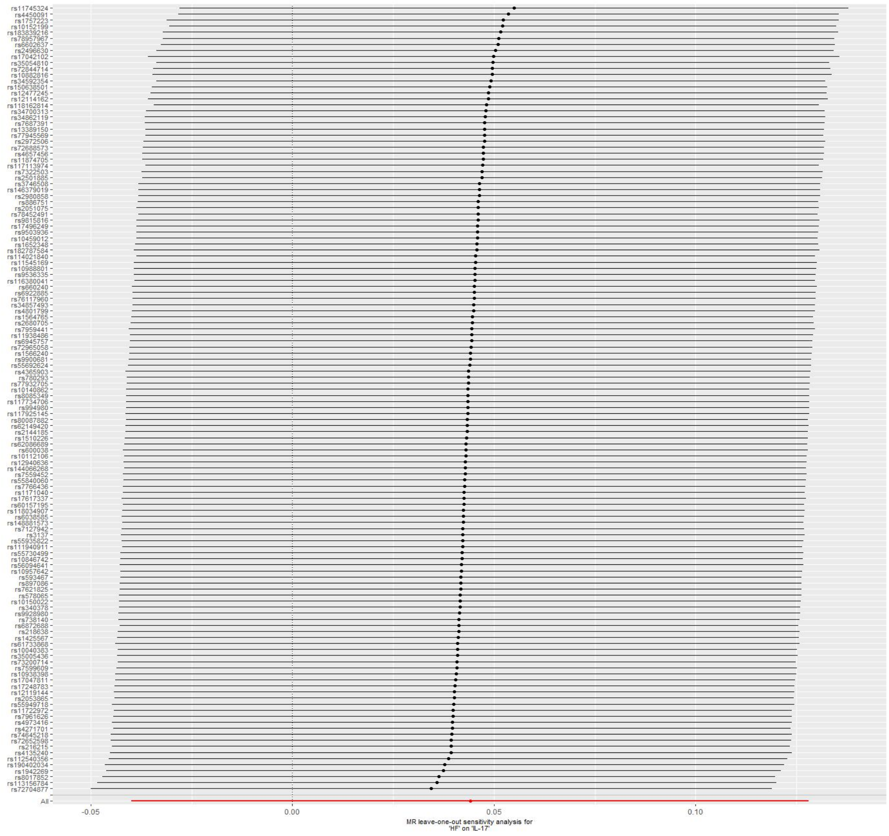

F PDGF-BB

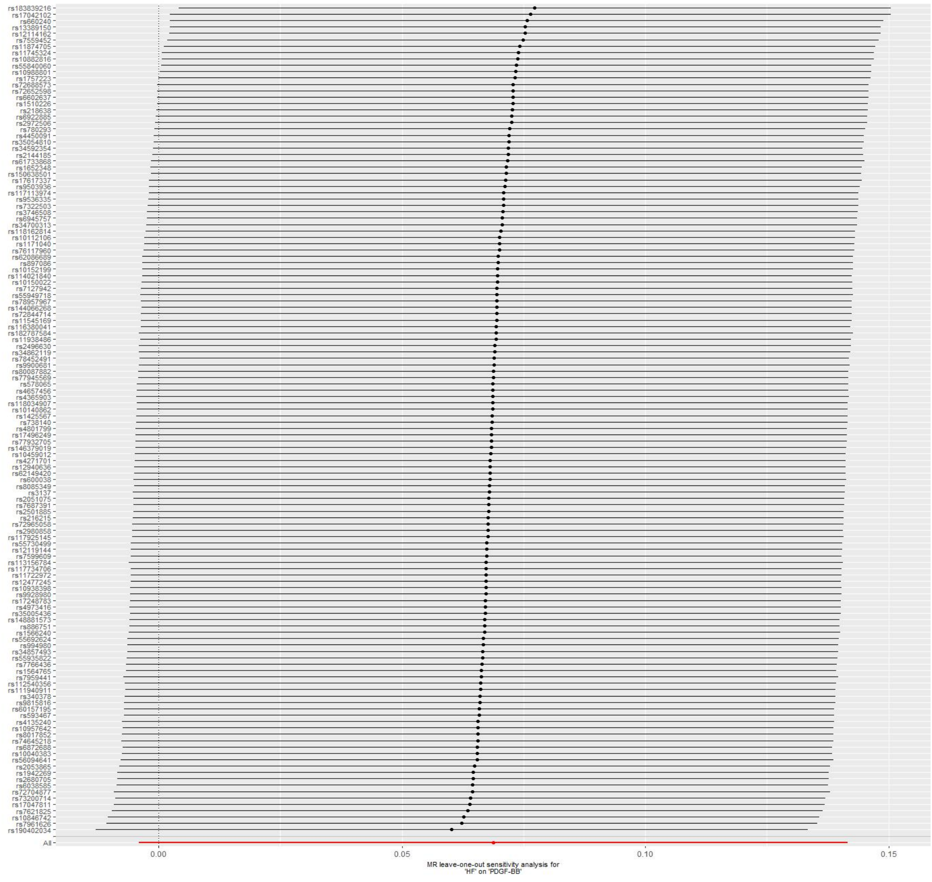

G FGF-basic

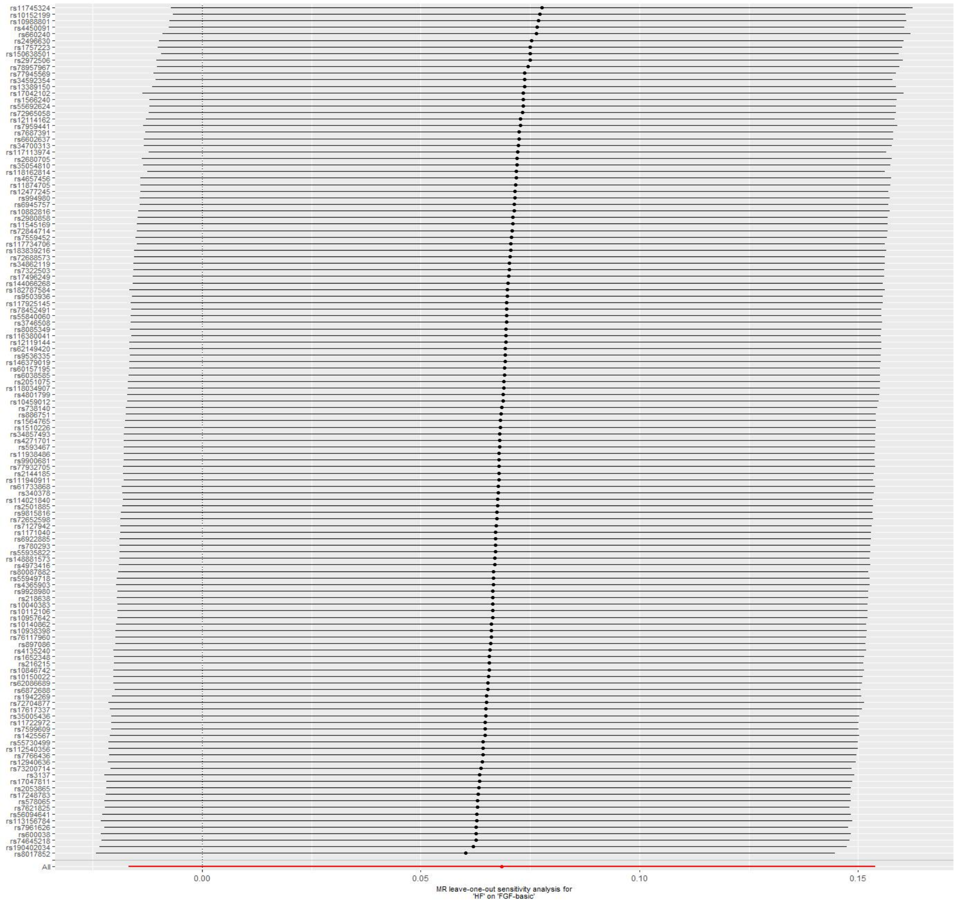
All
0.00
0.05
0.10
0.15
MR leave-one-out sensitivity analysis for
'HF' on 'FGF-basic'
